# Large-scale CSF proteome profiling identifies biomarkers for accurate diagnosis of frontotemporal dementia

**DOI:** 10.1186/s13024-025-00882-5

**Published:** 2025-08-27

**Authors:** Yanaika S. Hok-A-Hin, Lisa Vermunt, Carel F.W. Peeters, Emma L. van der Ende, Sterre C.M. de Boer, Lieke H. Meeter, Julie de Houwer, Harro Seelaar, John C. van Swieten, William T. Hu, Alberto Lleó, Daniel Alcolea, Sebastiaan Engelborghs, Anne Sieben, Alice Chen-Plotkin, David J. Irwin, Wiesje M. van der Flier, Yolande A.L. Pijnenburg, Charlotte E. Teunissen, Marta del Campo

**Affiliations:** 1 Neurochemistry Laboratory, Department of Laboratory Medicine, Amsterdam Neuroscience, VU University Medical Center, Amsterdam UMC, Amsterdam, The Netherlands; 2 Alzheimer Center, Department of Neurology, Amsterdam Neuroscience, VU University Medical Center, Amsterdam UMC, Amsterdam, The Netherlands; 3 Mathematical & Statistical Methods group – Biometris, Wageningen University & Research, Wageningen, The Netherlands; 4 School of Psychology and Brain & Mind Centre, The University of Sydney, Sydney, Australia; 5 Alzheimer Center and Department of Neurology, Erasmus Medical Center, Rotterdam, The Netherlands; 6 Department of Neurology, Center for Neurodegenerative Diseases Research, Emory University School of Medicine, Atlanta, USA; 7 Department of Neurology, Institut d’Investigacions Biomèdiques Sant Pau (IIB SANT PAU) - Hospital de Sant Pau, Universitat Autònoma de Barcelona, Hospital de la Santa Creu i Sant Pau, Barcelona, Catalunya Spain; 8 Center of Biomedical Investigation Network for Neurodegenerative Diseases (CIBERNED), Madrid, Spain; 9 Reference Center for Biological Markers of Dementia (BIODEM), Department of Biomedical Sciences, University of Antwerp, Antwerp, Belgium; 10 VrijeUniversiteit Brussel, Center for Neurosciences (C4N), Neuroprotection and Neuromodulation Research Group (NEUR), Vrije Universiteit Brussel, Brussels, Belgium; 11 Universitair Ziekenhuis Brussel, Department of Neurology, Brussels, Belgium; 12 Lab of Neuropathology, Neurobiobank, Institute Born-Bunge, Antwerp University, Edegem, Belgium; 13 Department of Neurology, Perelman School of Medicine, University of Pennsylvania, Philadelphia, PA USA; 14 Barcelonaßeta Brain Research Center, Pasqual Maragall Foundation, Barcelona, Spain; 15 Departamento de Ciencias Farmacéuticas y de la Salud, Facultad de Farmacia, Universidad San Pablo-CEU, CEU Universities, Madrid, Spain

**Keywords:** FTD, FTLD, Tau, TDP43, Biomarkers, Proteomics, CSF

## Abstract

**Background:**

Diagnosis of Frontotemporal dementia (FTD) and its specific underlying neuropathologies (frontotemporal lobar degeneration; FTLD-Tau and FTLD-TDP) are challenging, and thus, fluid biomarkers are needed to improve diagnostic accuracy.

**Methods:**

We used proximity extension assays to analyze 665 proteins in cerebrospinal fluid (CSF) samples from a multicenter cohort, which included patients with FTD (*n* = 189), Alzheimer’s Disease dementia (AD; *n* = 232), and cognitively unimpaired individuals (*n* = 196). In a subset, FTLD neuropathology was determined based on phenotype or genotype (FTLD-Tau = 87 and FTLD-TDP = 67). Differences in protein expression profiles were analyzed using nested linear models. Penalized generalized linear modeling was used to identify classification protein panels, which were translated to custom multiplex assays and validated in two clinical cohorts (cohort 1: *n* = 161; cohort 2: *n* = 162), one autopsy-confirmed cohort (*n* = 100), and one genetic cohort (*n* = 55).

**Results:**

Forty-three proteins were differentially regulated in FTD compared to controls and AD, reflecting axon development, regulation of synapse assembly, and cell-cell adhesion mediator activity pathways. Classification analysis identified a 14- and 13-CSF protein panel that discriminated FTD from controls (FTD diagnostic panel, AUC: 0.96) or AD (FTD differential diagnostic panel, AUC: 0.91). Custom multiplex panels confirmed the strong discriminative performancen between FTD and controls (AUCs > 0.96) and between FTD and AD (AUCs > 0.88) across three validation cohorts, including one with autopsy confirmation (AUCs > 0.90). Validation in genetic FTD (including *C9orf72*,* GRN*, and *MAPT* mutation carriers) revealed high accuracy of the FTD diagnostic panel in identifying both the presymptomatic (AUCs > 0.95) and symptomatic (AUC: 1) stages. Six proteins were differentially regulated between FTLD-TDP and FTLD-Tau. However, a reproducible classification model could not be generated (AUC: 0.80).

**Conclusions:**

Overall, this study introduces novel FTD-specific biomarker panels with potential use in diagnostic settings.

**Supplementary Information:**

The online version contains supplementary material available at 10.1186/s13024-025-00882-5.

## Background

Frontotemporal dementia (FTD) is the second most common form of young-onset dementia (i.e., defined as dementia with symptom onset before the age of 65). FTD presents heterogeneously, comprising various clinical, neuropathological, and genetic forms. From a clinical perspective, FTD can present with either behavioral and social changes (behavioral variant FTD, bvFTD), language impairment (semantic variant primary progressive aphasia, svPPA; and non-fluent variant PPA, nfvPPA), or motor dysfunction (corticobasal syndrome, CBS; and progressive supranuclear palsy, PSP) [1–4]. FTD is caused by different underlying neuropathologies leading to frontal temporal lobar degeneration (FTLD). Approximately 50% of FTLD cases develop aggregates of the microtubule Tau protein (FTLD-Tau), while 45% are characterized by cytoplasmic inclusion of the TDP-43 protein (FTLD-TDP). A small percentage of FTLD cases (5%) develop aggregates of the FUS protein (FTLD-FET) [5–7]. These pathologies can be predicted in familial forms of FTLD, where autosomal dominant mutations in the microtubule-associated protein tau (*MAPT)* gene lead to FTLD-Tau pathology, while mutations in the progranulin (*GRN)* or chromosome 9 open reading frame 72 (*C9orf72)* genes are associated with FTLD-TDP pathology [5, 8]. However, genetic FTD accounts for only 10–20% of FTLD cases, and in many families, the mutation status remains unknown, and novel genetic variants continue to be identified [9–12]. In sporadic cases, which are more common, the different neuropathological subtypes poorly correlate with the clinical presentation, with the exception of FTD accompanied with motor neuron disease and svPPA that shows a clinicopathological correlation with TDP-43 [13]. Thus, the different clinical phenotypes can have overlapping pathologies despite diverse genetic backgrounds, stressing the complexity of the disease and the importance of developing pathology-specific biomarkers, which are needed for targeted treatment.

Currently, there are no fluid biomarkers that specifically capture underlying FTD pathogenesis [14]. Given the overlapping clinical features with Alzheimer’s disease (AD) [15, 16], the analysis of the core AD-CSF biomarkers (i.e., amyloid-beta 1–40 and 1–42, Aβ1–40 and Aβ1–42 or the Aβ1–42/Aβ1–40 ratio; phosphorylated Tau181, pTau181; and total Tau, tTau) is often used to exclude AD diagnosis [17]. The concentrations of Neurofilament light (NfL) in CSF and blood are strongly increased in FTD but also in other neurodegenerative disorders since it reflects neuroaxonal damage [18, 19]. Nevertheless, this biomarker is considered valuable for distinguishing FTD from non-neurodegenerative disorders, such as primary psychiatric diseases, and it also shows good prognostic potential [20, 21]. Several studies have shown that the CSF pTau/tTau ratio could demarcate the main FTLD subtypes but with limited diagnostic accuracy [22, 23]. However, promising findings have been recently obtained with plasma GFAP/NfL ratio (FTLD-Tau vs. FTLD-TDP; AUC: 0.89), which requires additional validation in external cohorts [24]. Overall, there is an unmet need for fluid biomarkers specifically identifying FTD and its underlying pathologies, which is essential for accurate diagnosis, clinical trial inclusion, and to monitor the effects of treatments [25].

CSF proteomics offer the opportunity to study brain changes and associated processes in vivo. As extensively explored in AD, such analysis could offer insights into the disease’s etiology and reveal novel biomarker candidates [26–29]. Previous FTD biomarker discovery studies using unbiased mass spectrometry (MS)-based technologies identified multiple biomarker candidates (e.g., YKL-40 in FTD and NPTXR in *GRN* mutation carriers) [30, 31]. However, subsequent validation efforts did not show sufficient diagnostic accuracy of these markers [32–35]. The clinical and pathological heterogeneity of FTD and low sample sizes could explain the lack of validated biomarkers [30, 31]. In addition, the translation of novel FTD biomarkers could be hampered due to the use of different technologies between the discovery (e.g., MS) and validation (immunoassay-based methods) phases. Therefore, we followed a similar strategy to that previously applied for AD and dementia with Lewy bodies (DLB) [29, 36]. We here used the high-throughput immune-based proximity extension assay (PEA) to analyze the CSF proteome of an extensive and well-characterized cohort of patients with FTD presenting at memory clinics. We first aimed to identify CSF protein changes specifically associated with FTD and its main pathological subtypes (i.e., FTLD-Tau and FTLD-TDP). Next, we aimed to translate these findings into clinically feasible CSF protein panels to discriminate FTD and its pathological subtypes. Finally, we validated these panels in four independent cohorts, including an autopsy-confirmed and a genetic cohort.

## Method

### Participants

As part of our previous work [29], the discovery cohort (total = 617; Table 1) included CSF samples from patients diagnosed with FTD (*n* = 189), AD dementia (*n* = 232), and cognitively normal individuals (CON, *n* = 196). Most of the samples were selected from the Amsterdam Dementia Cohort (ADC; 120 FTD, 214 AD, 190 CON) [37]. To enrich for samples from patients with confirmed FTD, additional cases from the Center for Neurodegenerative Disease Research at the University of Pennsylvania (Penn; 46 FTD, 18 AD, 6 CON), Erasmus Medical Center (19 FTD), and the Goizueta Alzheimer’s Disease Research Center at Emory University (4 FTD) were selected. For a subset of the FTD patients (82%), the underlying neuropathology was known or could be predicted based on specific clinical diagnosis (FTLD-Tau = 87, FTLD-TDP = 67). FTLD-Tau was confirmed based on autopsy (*n* = 17), *MAPT* mutation (*n* = 16), and further enriched with patients clinically diagnosed with PSP (*n* = 31) and CBS (*n* = 23), which primarily associates with Tau neuropathology [38]. The FTLD-TDP group included autopsy-confirmed cases (*n* = 25) and patients with *GRN* (*n* = 8) or *C9orf72* (*n* = 23) mutations, and further enriched with patients clinically diagnosed with svPPA (*n* = 11), which have a high likelihood of having TDP pathology [13]. Four additional validation cohorts were included for validation of the custom panels: two clinical cohorts, from the ADC (51 FTD, 55 AD, and 55 controls) and Sant Pau Initiative on Neurodegeneration (SPIN; 54 FTD, 53 AD, and 55 controls) [39], one FTLD/AD autopsy confirmed cohort from the Reference Center for Biological Markers of Dementia (BIODEM) and the neurobiobank of the Institute Born-Bunge (IBB) from the University of Antwerp (UAntwerp) (BIODEM-UAntwerp; 41 autopsy-FTLD; aFTLD, including 7 aFTLD-Tau and 31 aFTLD-TDP, 3 aFTLD-UPS, and 30 autopsy-AD; aAD). Noteworthy, 29 cognitively unimpaired controls from BIODEM-UAntwerp were included in this cohort, though not autopsy-confirmed. Lastly, individuals from the Frontotemporal Dementia Risk Cohort (FTD RisC) at Erasmus Medical Center were included. This cohort comprises patients with FTD caused by autosomal dominant mutations (14 symptomatic mutation carriers, including 10 *C9orf72*, 3 *GRN*, and 1 *MAPT*). Additionally, their at-risk relatives were also included, consisting of 8 non-carriers and 33 presymptomatic mutation carriers (22 *C9orf72*, 7 *GRN*, and 4 *MAPT*).

**Table 1:** Cohort characteristics

	Discovery Cohort			Validation Cohort 1			Validation Cohort 2			FTLD/AD neuropathological		
	**Control**	**FTD**	**AD**	**Control**	**FTD**	**AD**	**Control**	**FTD**	**AD**	**Control**	**aFTLD**	**aAD**
n	196	189	232	55	51	55	55	54	53	29	41	30
FTD Clinical Phenotype		35 bvFTD			38 bvFTD			31 bvFTD			na	
		23 CBS			0 CBS			18 CBS-PSP*			na	
		31 PSP			0 PSP			1 lvPPA			na	
		11 svPPA			5 svPPA			2 svPPA			na	
		0 nfvPPA			2 nfvPPA			2 nfvPPA			na	
		42 Autopsy-confirmed			0 Autopsy-confirmed			0 Autopsy-confirmed			41 Autopsy-confirmed	
		47 Mutation Carriers			6 Mutation Carrier			0 Mutation Carriers			na	
Sex, Female n (%)	72 (36.7)	93 (49)	96 (41)	22 (40)	17 (33)	23 (42)	32 (58)	21 (38)	34 (64)	13 (45)	20 (49)	15 (50)
Age	58 (53 - 63)^a,b^	65 (59 - 70)^c^	66 (59 - 72)^c^	58 (56 - 62)^a,b^	63 (59 - 69)^c^	66 (62 - 70)^c^	61 (58 - 67)^a,b^	76 (71 - 79)^c^	74 (68 - 76)^c^	64 (61 - 66)^b^	64 (55 - 71)^b^	72 (64 - 76)^a,c^
MMSE	28 (27 - 29)^a,b^	25 (22 - 27)^b,c^	21 (17 - 24)^a,c^	29 (27 - 30)^a,b^	25 (23 - 27)^b,c^	20 (18 - 23)^a,c^	29 (29 - 30)^a,b^	21 (18 - 25)^c^	22 (19 - 25)^c^	29.5 (29 - 30)	20 (14 - 26)	14 (7 - 21)
CSF Aβ42, pg/mL	1120 (1023 - 1241)^a,b^	985 (782 - 1193)^b,c^	602 (535 - 657)^a,c^	1283 (1085 - 1373)^a,b^/ 1629 (1372 - 1700)^b^	1086 (888 - 1253)^b,c^/ 1336 (1038 - 1634)^b^	566 (525 - 661)^a,c^/ 489 (468 - 576)^a,c^	1194 (899 - 1397)^a,b^	871 (591 - 1268)^b,c^	537 (441 - 646)^a,c^	1108 (952 - 1216)^a,b^	681 (494 - 866)^b,c^	404 (306 - 497)^a,c^
CSF pTau, pg/mL	38 (31 - 45)^a,b^	44 (36 - 57)^b,c^	92 (77 - 112)^a,c^	54 (39 - 66)/ 15 (12 - 19)^b^	46 (39 - 61)^b^/ 17 (12 - 24)^b^	65 (50 - 92^)a^/ 35 (31 - 37)^a^	38 (28 - 47)^b^	41 (32 - 49)^b^	117 (81 - 165)^a,c^	47 (39 - 56)^b^	39 (29 - 49)^b^	72 (49 - 84)^a,c^
CSF tTau, pg/mL	213 (167 - 257)^a,b^	324 (239 - 414)^b,c^	751 (581 - 1005)^a,c^	292 (213 - 443)^b^/ 182 (150 - 230)	362 (254 - 487)^b^/ 230 (174 - 290)	479 (343 - 684)^a^/ 336 (327 - 346)	257 (198 - 314)^b^	324 (224 - 434)^b^	740 (546 - 976)^a,c^	203 (168 - 257)^a,b^	330 (222 - 441)^b,c^	535 (325 - 674)^a,c^
APOE4 carrier, n (%)	48 (25)	46 (24)	132 (57)	15 (27)	13 (26)	39 (71)	14 (26)	8 (15)	22 (41.5)	na	na	na
FTLD-Subtypes		FTLD-Tau (87 total):			na			na			aFTLD-Tau (7 total)	
		17 Autopsy-confirmed									aFTLD-TDP (31 total)	
		16 *MAPT*									aFTLD-UPS (3 total)	
		31 PSP										
		23 CBS										
		FTLD-TDP (67 total):										
		25 Autopsy-confirmed										
		8 *GRN*										
		23 *C9orf72*										
		11 svPPA										

Continuous variables are presented as median ± interquartile range and dichotomous data as the number of cases with a percentage of the total (%)

Differences between groups were determined using the Kruskal-Wallis test with Bonferroni correction or Chi-squared test

Analysis of covariance was performed for CSF biomarker analysis, adjusting for age and sex when appropriate

In the discovery cohort, biomarker values from the luminex were transformed to innotest values using passingbablok regression formulas (method)

For validation cohort 1, the first set of biomarker values corresponds to data obtained using Innotest, while the second set corresponds to data obtained using Elecsys

CSF Biomarker data from validation cohort 2 were obtained with Lumipulse

a, p< 0.05 compared to FT(L)D

b, p< 0.05 compared to AD

c, p< 0.05 compared to CON

* 18 Patients were on the spectrum of CBS and PSP, with 11 showing a clinical profile more consistent with CBS, and 4 exhibiting features more indicative of PSP

Abbreviations: FTD = frontotemporal dementia, AD = Alzheimer’s Disease, MMSE = mini-mental state examination, CSF = cerebrospinal fluid,

FTLD = frontotemporal lobar degeneration, na = not applicable, TDP = Transactive response DNA binding protein of 43, FUS = fused in sarcoma, aFTLD = autopsy confirmed FTLD, aAD = autopsy confirmed AD

All participants underwent standard neurological screening and cognitive testing. FTD and AD diagnoses were made according to international consensus criteria [1–4, 40]. The autopsy-confirmed cohort included cases with a definite diagnosis according to international neuropathological examination guidelines for FTLD [41] and AD [42]. The genetic FTD cohort included participants identified as symptomatic based on international guidelines [1, 2]. Symptomatic onset was defined as the point at which caregivers first reported the presence of symptoms. Mini-mental state examination (MMSE) was used as a measurement for global cognition in all groups. In addition, for a subset of FTD cases from the ADC (discovery cohort, *n* = 62; clinical cohort 1, *n* = 44), the FTLD CDR^®^ plus NACC score was used as a clinical measure specific for FTD disease severity [43]. The control group included individuals with subjective cognitive decline, who scored normally on cognitive examinations with a negative AD CSF biomarkers profile.

CSF was collected and biobanked according to established protocols [44]. Concentrations of CSF Aβ42, pTau, and tTau were used to support AD diagnosis and to evaluate the presence of AD co-pathology in the FTD cases. These were analyzed locally as part of the diagnostic work-up using commercially available kits (ADC, Erasmus MC, and BIODEM-UAntwerp: ELISA Innotest Aβ(1-42), hTAUAg, pTau (181P; Fujirebio, Ghent), or ADC: Elecsys Aβ42, tTau and pTau (181P) CSF assays (Roche Diagnostics); Penn and Emory: Luminex xMAP INNO-BIA AlzBio3 (Luminex, Bio-techne); SPIN: Lumipulse G600, Fujirebio). Predefined cut-offs were used to define a positive AD biomarker profile. (ADC: tTau/Aβ42 ratio > 0.46; Penn: tTau/Aβ42 ratio > 0.30; Emory: Aβ42/tTau ratio < 6; SPIN: Aβ42/Aβ40 ratio < 0.062, tTau > 456 pg/mL and pTau > 63 pg/mL). In the ADC, Innotest Aβ42 concentrations were adjusted for drift over time [45]. CSF biomarkers measured on Luminex were transformed to Innotest values using conversion formulas based on Passing-Bablok regression analysis using cases for which both Luminex and Innotest values were available, as described previously [29]. CSF NfL was measured in a subset of cases from the ADC (discovery cohort: 42 CON, 74 FTD, and 54 AD; clinical cohort 1: 8 CON, 19 FTD, and 20 AD) either by NF-light^®^ ELISA (Uman Diagnostics, Sweden) or with the single molecular array (Simoa^®^) NF-light™ advantage kit (Quanterix, USA) [46]. NfL concentrations measured by ELISA were converted to Simoa values using Passing-Bablok regression analysis, as described previously [46].

### CSF protein profiling

979 CSF proteins were quantified using 11 Olink Target 96 validated multiplex panels based on proximity extension assay (PEA) technology (Olink Proteomics, Uppsala, Sweden) that were available at the time of analysis (Cardiometabolic, Cardiovascular II and III, cell regulation, development, immune response, inflammation, metabolism, neurology, oncology II and organ damage). CSF samples were randomized across multiple plates containing intra- and inter-plate quality controls (QCs) from the manufacturer and measured in two different rounds. Each round of measurement contained 16 bridging samples covering different clinical groups, which were used as a reference to account for potential batch effects. For each protein, the lower limit of detection (LOD) was determined by the company and defined as three standard deviations above the background from the negative controls included on every plate. Proteins were excluded from further analysis if their levels were below the LOD in more than 15% of the samples. A total of 665 proteins (642 unique proteins) were ultimately included for statistical analysis of the discovery cohort, similar to our previous study [29]. Protein abundance was reported in normalized protein expression (NPX) values.

### Development of custom PEA assays

Multiplex-PEA assays were custom-developed by the manufacturer following standardized protocols [47]. We developed assays to measure 18 out of 24 proteins selected by the classification analysis described below. CSF samples from validation cohorts were randomized across plates. Each plate additionally included: four CSF QC samples, a negative control, and three calibrators used for normalization. Each custom assay had a LOD determined by the company, defined as three standard deviations above the background from the negative controls. Precision (intra- and inter-assay CV) was calculated using the four CSF QC samples (Supplementary Table 1). No cross-reactivity between assays for the specific proteins was detected. Samples from the validation cohorts were randomized across plates and normalized for any plate effects using the built-in inter-plate controls according to the manufacturer’s recommendations. Protein levels are reported in NPX values.

### Statistical analyses

All processing and statistical analysis were performed in R version 4.2.1. Baseline demographics were tested by Kruskal-Wallis test followed by Bonferroni post-hoc, or Peasons’s chi-square test for continuous and categorical variables, respectively. Differences in protein abundance of the CSF proteome data was tested with nested linear models including age and sex in the model for the comparison between different groups (FTD vs. controls, FTD vs. AD, FTLD-Tau vs. controls, FTLD-TDP vs. controls, and FTLD-Tau vs. FTLD-TDP) as previously performed [29, 36]. For each pairwise comparison, multiplicity was taken into account by controlling the False Discovery Rate (FDR) at *q* ≤ 0.05 based on the number of features analyzed [48].

We next evaluated which CSF protein combination (CSF panels) could best discriminate the groups of interest while keeping the number of markers to a minimum so that they can be ultimately translated into small, practical custom panels, as described previously [29]. For this purpose, binary classification signatures (FTD vs. controls: FTD Diagnostic Panel, FTD vs. AD: FTD Differential Diagnostic Panel, and FTLD-Tau vs. FTLD-TDP: FTLD Subtype Panel) were constructed by way of penalized generalized linear modeling (GLM) with an elastic net penalty (a linear combination of lasso and ridge penalties) in the discovery cohort using the glmnet package, including age and sex as covariates in the model [29, 49, 50]. This penalty enables estimation in settings where the feature-to-sample ratio is too high for standard generalized linear regression. Moreover, it performs automatic feature decorrelation as well as feature-selection. For each classification exercise, we compare multiple models that reflect (a) a grid of values for the elastic-net mixing parameter, reflecting strong decorrelation to a pure logistic lasso regression, and (b) a grid of values reflecting the maximum number of proteins that may be selected under each model (21 markers maximum). The former grid (a) considers that we have little information on the collinearity burden in the data. The latter grid (b) considers that we want to keep the number of selected proteins relatively low for the future development of customized panels. The optimal penalty parameters in the penalized models were determined based on (balanced) 10-fold cross-validation of the model likelihood [29, 49]. The cross-validation was performed with balanced folds, by which each fold has an outcome group ratio close to the corresponding ratio in the full data set, also referred to as stratified cross-validation. The predictive performance of all models was assessed by way of (the comparison of) Receiver Operating Characteristic (ROC) curves and Area Under the ROC Curves (AUCs). The model with the highest AUC and lowest number of markers for each classification signature was selected. The fold-based selection proportions for each marker were assessed to identify and select the most promising markers within each model (i.e., features that are stably selected across each individual fold, thereby minimizing potential overfitting). To reflect the manual selection pressure for these final marker sets, each final logistic signature was subjected to a ridge-regularization with a penalty parameter of 0.1. The performance (AUC) was evaluated by internal validation: repeated 5-fold cross-validation with 1000 repeats. The 95% confidence interval around the resulting AUCs was based on resampling quantiles (percentile method). External validations assessed the performance of the final models with the markers of interest in the validation cohorts. We additionally compared our identified classification models to CSF NfL to discriminate FTD from controls and AD in the subset of cases for which this information was available.

Functional enrichment analysis was performed for all comparisons described above using Metascape, selecting GO biological Processes as an ontology source [51]. The total number of CSF proteins optimally analyzed was included as the enrichment background (*n* = 642). Default parameters were used for the analysis in which terms with a *p*-value < 0.01, a minimum count of 3, and an enrichment factor > 1.5 were collected and grouped into clusters based on their membership similarities.

Partial non-parametric correlation analysis was performed to understand the association among proteins within the CSF panels with cognitive function (MMSE score) and disease severity (FTLD-CDR^®^ plus NACC scores) [43]. This analysis was corrected for age, sex, and the clinician who performed the FTLD-CDR examination (included as a dummy variable), and was conducted both in the total discovery cohort and specifically within the FTD group.

## Results

### Subjects

**Fig. 1:**
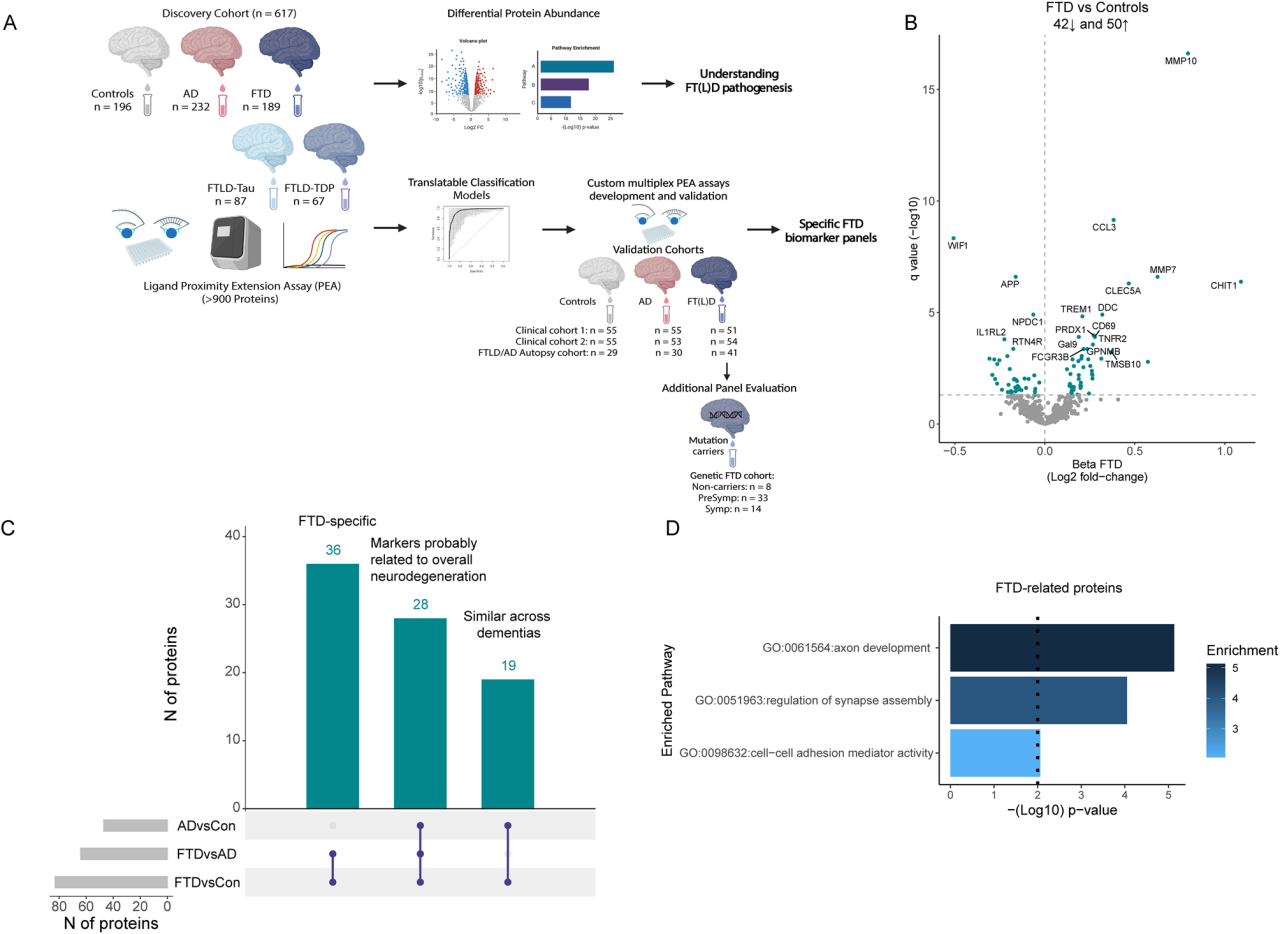
Overview study design and differential abundance of CSF proteins in FTD. (**A**) More than 900 proteins were measured using the antibody-based PEA technology in CSF from 196 cognitively unimpaired controls (white), 189 FTD (blue), and 232 AD (red) patients. Differential protein abundance was investigated and classification models were constructed. Custom PEA assays using the proteins identified in the classification models were developed and validated in four independent cohorts, including an FTLD/AD autopsy cohort and genetic cohort. (**B**) Volcano plots show that 93 CSF proteins were differentially regulated between FTD and controls. Each dot represents a protein. The beta coefficients (log2 fold-change) are plotted versus q values (-log10-transformed). Proteins significantly dysregulated after adjusting for false discovery rate (FDR, q < 0.05) are depicted in blue. The total number of proteins that are down-regulated (*n* = 42, left) or up-regulated (*n* = 51, right) are depicted. Horizontal dotted line indicates the significance threshold. (**C**) UpSet plot shows proteins dysregulated between FTD and controls and also dysregulated between FTD and AD or AD and controls. (**D**) Bar graphs depicting the biological pathways enriched in protein specifically dysregulated in FTD. The dotted line represents the significant threshold (*p* < 0.01). The corresponding GO number and biological process is depicted on the left side. Stronger colors represent higher significant enrichment

An overview of the study is presented in Fig. 1A. The discovery cohort included a total of 617 samples, all measured as part of previous CSF proteomic studies [29, 36]. Custom multiplex panels were developed and validated in two independent clinical cohorts (validation cohort 1: *n* = 161; validation cohort 2: *n* = 162), one FTLD/AD autopsy-confirmed cohort (*n* = 100), and one genetic FTD cohort (*n* = 55). The demographic characteristics for the FT(L)D and AD cohorts are described in Table 1 and the genetic FTD cohort is described in Supplementary Table 1. Controls were younger compared to other diagnostic groups in the discovery cohort, clinical cohort 1, and clinical cohort 2. Patients with AD were older, and patients with FTLD were overall younger in the FTLD/AD autopsy cohort compared to the other cohorts. In the genetic FTD cohort, presymptomatic individuals were significantly younger compared to the symptomatic group (Supplementary Table 1).

### CSF proteins are differentially regulated in FTD compared to controls and AD

CSF proteome profiling revealed 92 proteins differentially regulated between FTD and controls, of which 42 proteins were decreased and 50 proteins were increased (*q* < 0.05; Fig. 1B, Supplementary Table 2). The top 5 differentially regulated proteins related to FTD (median *q*: 2.5^− 07^) are involved in the regulation of the Wnt signaling pathway (WIF1), tissue remodeling (MMP7), neuronal biosynthesis (APP), cell proliferation (NPDC1), and innate immunity (IL1RL2). Sensitivity analysis showed that excluding FTD patients with AD co-pathology (*n* = 19, 10%) had minimal effect on the CSF proteome profiling results (Supplementary Fig. 1). Next, we sought to determine whether the protein changes identified were especially related to FTD. We show that 36 proteins (40%) were uniquely dysregulated in FTD compared to controls and AD (e.g., WIF1, ROBO2, and SLITRK2; Fig. 1C). Nineteen proteins (20%) were dysregulated in FTD and AD compared to controls, which likely represent general dementia processes (e.g., CHIT1, MMP10, and MMP12; Fig. 1C). Twenty-eight proteins (30%) were dysregulated across all comparisons: controls versus FTD and AD, and FTD versus AD. Most of these proteins (21 out of 28; e.g., MIF, ITGB2, and sTREM1) were to some extent increased in FTD but were more prominently dysregulated in AD, indicating that they are likely more related to AD pathophysiological processes. However, 7 out of the 28 proteins (e.g., CHL1, GPC1, CNTN5, HBEGF, CLSTN2, GCP5) showed an opposite direction of change in FTD and AD and are likely differently involved in the pathophysiology of these types of dementias. Functional enrichment analysis of the proteins related to FTD pathophysiology (i.e., those that were increased in FTD as well as those that were specifically dysregulated in FTD compared to AD, *n* = 43) revealed associations with axon development, regulation of synapse assembly, and cell-cell adhesion mediator activity (Fig. 1D).

### CSF protein panels discriminate with high accuracy FTD from controls and AD

**Fig. 2:**
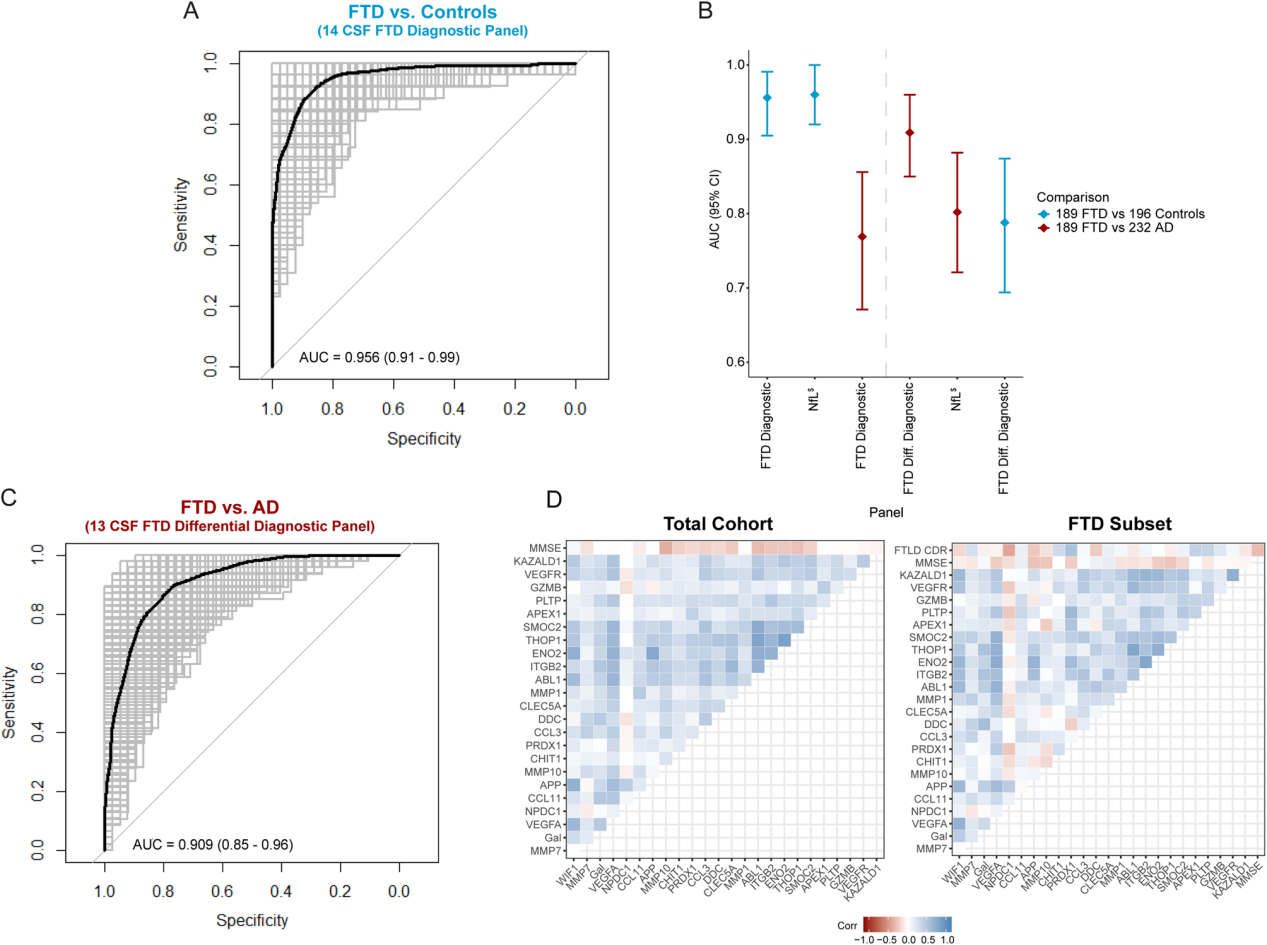
CSF biomarker panels for the diagnosis of FTD. (**A**) Receiver operating characteristic (ROC) curves depict the performance of 14-CSF biomarker panel discriminating FTD (*n* = 189) from controls (*n* = 196). Black line is the mean area under the curve (AUC) overall re-samplings (1000 repeats of 5-fold cross-validation, grey lines). (**B**) Forest plot shows the different AUC and 95% confidence interval for the 14 and 13 CSF biomarker panels and NfL to discriminate between FTD and controls (blue) or AD (red). (**C**) ROC curves depict the performance of 13-CSF biomarker panel discriminating FTD (*n* = 189) from AD (*n* = 232). (**D**) Correlation matrix heatmap representing the Spearman’s correlation coefficient in-between the proteins selected in each panel, MMSE score, and FTLD-CDR scores in the total cohort, and in a subset of the FTD group (*n* = 62)

Next, we aimed to identify CSF biomarkers specific for FTD. One of the strongest proteins specifically dysregulated in FTD was WIF1, however, its performance as a single marker to discriminate FTD from controls was moderate (AUC: 0.794, 95% CI: 0.75–0.84). Thus, we next performed a classification analysis, followed by internal cross-validation, to investigate whether a combination of proteins could discriminate FTD from controls with higher accuracy (CSF panels, Fig. 1A). We identified a panel of 14 CSF proteins that discriminated FTD from controls with high accuracy (FTD diagnostic panel; AUC: 0.96, 95% CI: 0.91–0.99; Fig. 2A), which was comparable to the performance of CSF NfL (AUC: 0.96, 95% CI: 0.92-1; Fig. 2B). The model included proteins that were dysregulated specifically in FTD (i.e., WIF1, MMP7, GAL, VEGFA, NPDC1, APP, and CCL11; Supplementary Fig. 2), as wel as proteins related to common neurodegenerative processes and other neurodegenerative dementias (i.e., MMP1, MMP10, CHIT1, CCL3, PRDX1, and DDC; Supplementary Fig. 2), or reported to be associated with AD (i.e., CLEC5A; Supplementary Fig. 2). The performance of the FTD diagnostic panel to discriminate FTD from AD was considerably lower (FTD diagnostic panel AUC: 0.77, 95% CI: 0.67–0.86; for comparison: CSF NfL AUC: 0.80, 95% CI: 0.72–0.88, Fig. 2B). We thus next investigated whether we could find a combination of proteins that optimally discriminated FTD from AD. A panel consisting of 13 CSF proteins was identified that discriminated FTD from AD with high accuracy (FTD differential diagnostic panel; AUC: 0.91, 95% CI: 0.85–0.96; Fig. 2C). This panel contained proteins associated with FTD (i.e., GZMB, MMP7, CCL11, NPDC1, PLTP, and APEX1; Supplementary Fig. 3) but also proteins associated with AD (i.e., ABL1, ENO2, ITGB2, SMOC2, and THOP1; Supplementary Fig. 3), as also reported in our previous AD focused PEA study [29]. The model also included two proteins with no differences across groups, likely due to the model’s adjustment for inter-individual variability (i.e., VEGFR3 and KAZALD1; Supplementary Fig. 3) [52]. Of note, three proteins (i.e., MMP7, NPDC1, and CCL11) were included in both the FTD diagnostic and FTD differential diagnostic panel. The FTD-related proteins from these panels are associated with diverse biological pathways including inflammatory processes (GZMB), tissue remodeling (MMP1 and MMP7), cell proliferation (NPDC1), oxidative stress (APEX1), vascular functioning (VEGFA), and neuronal biosynthesis and functioning (APP and GAL) [53–57].

Next, we sought to determine if the proteins included in our FTD panels are associated with disease severity and cognition as measured by FTLD-CDR and MMSE scores. In the FTD patients, we observed that PRDX1 (*Rho* = 0.47, *p* < 0.001) had a moderate to strong correlation while NPDC1 (*Rho* = -0.37, *p* < 0.001), APEX (*Rho* = 0.35, *p* < 0.01), PLTP (*Rho* = 0.33, *p* < 0.01), and CHIT1 (*Rho* = 0.28, *p* < 0.05) showed a moderate correlation with FTLD-CDR scores (Fig. 2D). In the total cohort, we show that the proteins associated with AD (ABL1, ENO2, ITGB2, SMOC2, and THOP1) together with MMP10 had the strongest correlations with MMSE scores (*Rho’s* between − 0.19 and − 0.34, all *p* < 0.001; Fig. 2D).

### CSF proteins are differentially regulated in FTLD-Tau or FTLD-TDP subtypes

**Fig. 3:**
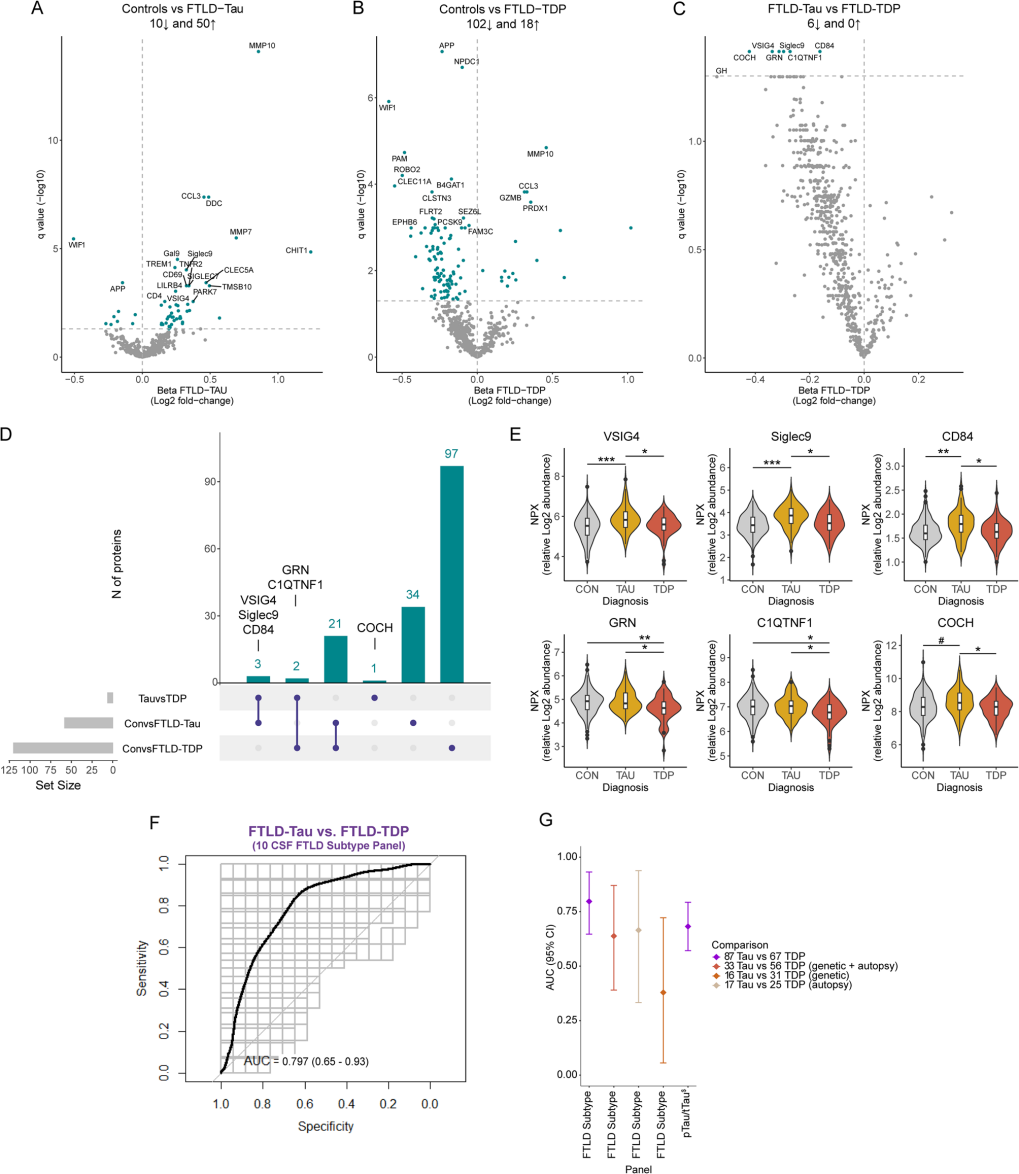
Differential abundance of CSF proteins in FTLD subtypes and FTLD biomarker panel. Volcano plots show CSF proteins differentially regulated between patients with FTLD-Tau (*n* = 87; **A**) or FTLD-TDP (*n* = 67; **B**) and controls (*n* = 196) and between these neuropathological subtypes (**C**). Each dot represents a protein. The beta coefficients (log2 fold-change) are plotted versus q values (-log10-transformed). Proteins significantly dysregulated after adjusting for false discovery rate (FDR, q < 0.05) are depicted in blue. The total number of proteins that are down-regulated (left) or up-regulated (right) is indicated. **D**) UpSet plot depicts proteins dysregulated between the FTLD-Tau, FTLD-TDP, and control groups. **E**) Violins represent the abundance (log2 NPX) of the CSF proteins that were uniquely dysregulated in FTLD-Tau and FTLD-TDP or between the subtypes. Boxplot within the violin indicates the median and interquartile range of the protein abundance. **F**) ROC curves depict the performance of 10-CSF biomarker panel discriminating FTLD-Tau (*n* = 87) from FTLD-TDP (*n* = 67). **G**) Forest plot shows the different AUC and 95% confidence interval for the 10 CSF biomarker panels, and pTau/tTau ratio to discriminate between neuropathological subtypes (purple; 87 FTLD-Tau and 67 FTLD-TDP), subtypes that are genetic and/or autopsy confirmed (brown; 33 FTLD-Tau and 56 FTLD-TDP), subtypes which are genetically confirmed (coral; 16 FTLD-Tau and 31 FTLD-TDP), and subtypes which are autopsy confirmed (beige; 17 FTLD-Tau and 25 FTLD-TDP). $ shows the comparison in a subset of cases (NfL: 76 FTD, 47 controls, and 54 AD; pTau/tTau ratio: 74 FTLD-Tau and 37 FTLD-TDP. # *p* < 0.05, *q < 0.05, **q < 0.01, ***q < 0.001. Abbreviations: CON, cognitively unimpaired controls; TDP, transactive response DNA binding protein 43

Our second aim was to investigate which protein dysregulations characterized the FTLD-Tau and FTLD-TDP subtypes. Compared to controls, we observed 60 proteins dysregulated in FTLD-Tau (top 5: MMP10, DDC, CCL3, MMP7, and WIF1; median *q* 4.2^− 08^, q < 0.05; Fig. 3A, Supplementary Table 3) and 120 proteins that were dysregulated in FTLD-TDP (top 5: APP, NPDC1, WIF1, B4GAT1, and ROBO2; median *q 1.2*^*− 06*^; Fig. 3B, Supplementary Table 4). When comparing FTLD-Tau to FTLD-TDP, we observed that 6 proteins (COCH, Siglec9, VSIG4, GRN, CD84, and C1QTNF1) were dysregulated between these groups (*q* < 0.05; Fig. 3C, Supplementary Table 5). This number increased to 185 dysregulated proteins when nominal significance was considered (i.e., *p* < 0.05, Supplementary Table 5). To show which proteins are specifically associated with one of the FTLD subtypes, we visualized the outcomes of three comparisons in an upset plot (Fig. 3D). Here, we identified three proteins uniquely related to FTLD-Tau (i.e., all increased in FTLD-Tau compared to controls and FTLD-TDP; Fig. 3E). These proteins play a role in various processes, including phagocytosis and the NLRP3 inflammasome (VSIG4), cell adhesion processes (Siglec9), and involvement in both the innate and adaptive immune responses (CD84) [58–60]. Two proteins were uniquely related to FTLD-TDP (i.e., both decreased in FTLD-TDP compared to controls and FTLD-Tau; Fig. 3E), which are related to lysosomal functioning (GRN) and the immune response (C1QTNF1) [61, 62]. Interestingly, one protein was uniquely dysregulated between FTLD-Tau and FTLD-TDP (COCH; Fig. 3E), with higher levels in FTLD-Tau. This protein plays a role in the modulation of cell shapes [63].

### CSF protein panel to discriminate the FTLD subtypes

We next sought to identify CSF biomarkers specific to the FTLD subtypes. A panel of 10 CSF proteins that discriminated between FTLD-Tau and FTLD-TDP with moderate performance was identified (AUC: 0.797, 95% CI: 0.65–0.93; Fig. 3F). Despite the performance being superior to the pTau/tTau ratio (AUC: 0.68, 95% CI: 0.57–0.79; Fig. 3G), the large CI reflects high variability in the cross-validation procedures and thus poor robustness. This model included proteins that were specifically dysregulated in FTLD-Tau (i.e., VSIG4, CD84, and Siglec9) or FTLD-TDP (i.e., GRN) and those that were nominally or significantly changed between subtypes (i.e., COCH, GH, CST5, and PRSS8; Supplementary Fig. 3), as well as proteins that were similar across groups (i.e., IFNLR1 and ANGPT2; Supplementary Fig. 4). The performance of this model did not improve after stratifying the FTLD groups for genetic or sporadic cases (AUCs: 0.37–0.64; Fig. 3G).

### Validation of FTD panels using custom PEA assays in independent cohorts

We next aimed to translate our proteomic findings into clinically useful tools. Thus, we developed custom multiplex-PEA assays containing 18 of the 24 proteins from the FTD panels described above (i.e., FTD diagnostic panel: 9 proteins; FTD differential diagnostic panel: 12 proteins; 3 proteins present in both panels). Six proteins could not be included due to technical limitations. The custom PEA assays (custom panels) showed over 90% detectability of the proteins in patient samples, with low coefficients of variation (CVs; <5% intra-CV and < 3% inter-CV; Supplementary Table 6). No custom multiplex-PEA assay was developed for the FTLD subtype panel.

**Fig. 4:**
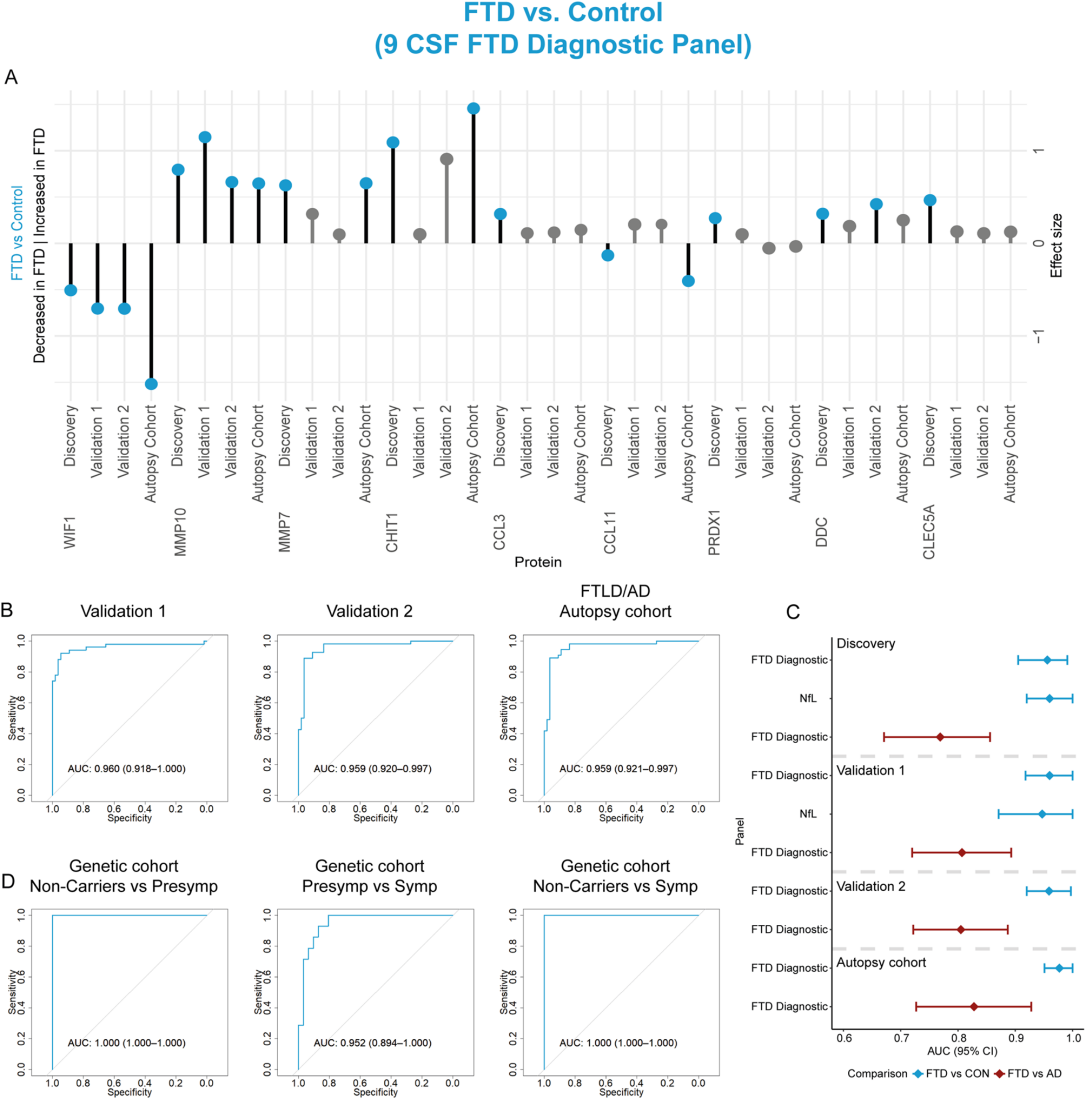
Development and validation of custom CSF biomarker panels for FTD diagnosis in independent cohorts. (**A**) Lollipop plots depict the beta-coefficients obtained in the discovery phase in parallel to the beta-coefficients of the custom assays in clinical validation cohorts 1 and 2 and the FTLD/AD autopsy cohort. Grey dots show proteins that did not remain significant after correction for multiple testing. (**B**) Receiver operating characteristic (ROC) curves showing the performance of the CSF biomarker panel discriminating FTD from controls using the custom assays across the two clinical and one autopsy validation cohort. Inserts outline corresponding AUC and 95% CI. (**C**) Forest plots depict the different AUC and 95% CI obtained with the CSF FTD biomarker panels or CSF NfL in the comparison between FTD and controls (blue) or AD (red). (**D**) ROC curves showing the performance of the FTD diagnostic panel in the FTD genetic cohort to discriminate non-carriers from presymptomatic (PreSymp) and symptomatic (Symp) mutation carriers, and presymptomatic from symptomatic

**Fig. 5:**
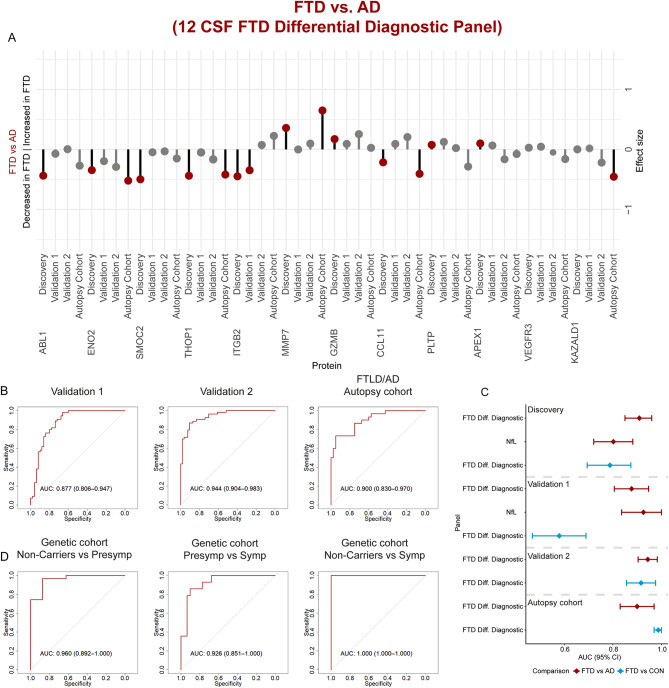
Development and validation of custom CSF biomarker panels for FTD differential diagnosis in independent cohorts. (**A**) Lollipop plots depict the beta-coefficients obtained in the discovery phase in parallel to the beta-coefficients of the custom assays in clinical validation cohorts 1 and 2 and the FTLD/AD autopsy cohort. Grey dots show proteins that did not remain significant after correction for multiple testing. (**B**) Receiver operating characteristic (ROC) curves showing the performance of the CSF biomarker panel discriminating FTD from AD using the custom assays across the two clinical and one autopsy validation cohort. Inserts outline corresponding AUC and 95% CI. (**C**) Forest plots depict the different AUC and 95% CI obtained with the CSF FTD biomarker panels or CSF NfL in the comparison between FTD and AD (red) or controls (blue). (**D**) ROC curves showing the performance of the FTD differential diagnostic panel in the FTD genetic cohort to discriminate non-carriers from presymptomatic (PreSymp) and symptomatic (Symp) mutation carriers, and presymptomatic from symptomatic

We compared the protein's fold changes obtained in the custom panels for three validation cohorts (including one with autopsy confirmation) to those in the discovery cohort. The protein´s fold changes obtained in the comparisons between FTD and controls significantly correlated between those detected in the discovery and two validation cohorts (clinical cohort 1: *Rho* = 0.45, *p* > 0.05, clinical cohort 2: *Rho* = 0.65, *p* < 0.05 and FTLD/AD autopsy cohort: *Rho* = 0.93, *p* < 0.001; Supplementary Fig. 5A). In addition, the fold changes of WIF1 and MMP10 were strong and replicated across all cohorts (Fig. 4A). Similarly, strong correlations of the protein fold changes for the difference between FTD and AD patients were observed between the discovery cohort and the three validation cohorts (clinical cohort 1: *Rho* = 0.69, *p* < 0.05; clinical cohort 2: *Rho* = 0.78, *p* < 0.01; FTLD/AD autopsy cohort: *Rho* = 0.87, *p* < 0.001, Supplementary Fig. 5B). Noteworthy, we observed that the fold changes of some proteins lost significance after correction for multiple testing in clinical cohort 1 and clinical cohort 2, whereas several proteins showed their strongest effects in the FTLD/AD autopsy cohort (e.g., THOP1, MMP7, ENO2, CCL11, and KAZALD1; Figs. 4A and 5A).

The FTD diagnostic panel (containing 9 out of the 14 proteins) confirmed discrimination of FTD from controls with high accuracy in all validation cohorts (clinical cohort 1: AUC = 0.96, 95% CI: 0.92-1; clinical cohort 2: AUC = 0.96, 95% CI: 0.92-1; FTLD/AD autopsy cohort: AUC = 0.98, 95% CI: 0.95-1; Fig. 4B) and was similar to CSF NfL (clinical cohort 1: AUC = 0.95, 95% CI: 0.87-1; Fig. 4C). Exclusion of the FTD patients with AD co-pathology (*n* = 8) in clinical cohort 1 had minimal impact on the performance of the FTD diagnostic panel (AUC = 0.960, 95% CI: 0.91-1; Supplementary Fig. 6A). In addition, this FTD diagnostic panel showed strong performance for discriminating symptomatic mutation carriers from non-carriers (AUC = 1, 95% CI: 1–1) and presymptomatic carriers (AUC = 0.95, 95% CI: 0.90-1) as wel as in differentiating presymptomatic carriers from non-carriers (AUC = 1, 95% CI: 1–1; Fig. 4D; Supplementary Fig. 7). The FTD differential diagnostic panel (containing 12 out of the 13 proteins) could again discriminate FTD from AD with high accuracy in the three validation cohorts (clinical cohort 1: AUC = 0.88, 95% CI: 0.81–0.95; clinical cohort 2: AUC = 0.94, 95% CI: 0.90–0.98; FTLD/AD autopsy cohort: AUC = 0.90, 95% CI: 0.83–0.97; Fig. 5B), which was similar to NfL (clinical cohort 1, AUC = 0.93, 95% CI: 0.84-1; Fig. 5C). Exclusion of the FTD patients with AD co-pathology (*n* = 8) in clinical cohort 1 increased the diagnostic performance of the FTD differential diagnostic panel (AUC = 0.939, 95% CI: 0.87–0.99; Supplementary Fig. 6B). In addition, the FTD differential diagnostic panel showed good performance for discriminating symptomatic mutation carriers from non-carriers (AUC = 1, 95% CI: 1–1) and presymptomatic carriers (AUC = 0.93, 95% CI: 0.86-1) as wel as in differentiating presymptomatic carriers from non-carriers (AUC = 0.96, 95% CI: 0.90-1; Fig. 5D; Supplementary Fig. 8). To summarize, we replicated the discriminative performance as we observed in the discovery cohort, both for the FTD diagnostic and differential diagnostic panel.

## Discussion

This study identified novel and specific protein changes for FTD and its main pathological subtypes. These findings were translated into two CSF biomarker panels that discriminated FTD from controls and AD patients with high accuracy (AUCs > 0.9). These results were validated by clinically feasible panels that measured the selected proteins in several independent cohorts, including an autopsy-confirmed and a genetic FTD cohort. We identified proteins specifically associated with FTLD-Tau or FTLD-TDP groups, but we could not identify a marker or combination of markers to robustly discriminate between these FTLD subgroups. Moreover, the panels identified the presymptomatic stage of FTD mutation carriers with high accuracy, while the further increase in the symptomatic stage suggests their potential as an objective tool for monitoring disease progression. The FTD proteins and biomarker panels identified in this study reflect a broad range of different biological processes associated with FTD and its biological subtypes including inflammatory processes, regulation of synapses, lysosomal functioning, tissue remodeling, and oxidative stress.

Fluid biomarkers specifically associated with FT(L)D pathophysiology are needed to improve diagnostic accuracy, for clinical trial inclusion, and to monitor treatment effects [14]. We have performed a large multicenter FTD proteomics study, including patients with different FTLD neuropathologies as well as a group with AD dementia. Up to 92 proteins were dysregulated between FTD and controls. By comparing the protein profiles to AD patients, we observed that 40% of these proteins were uniquely dysregulated in FTD (e.g., WIF1, ROBO2, and SLITRK2). Some proteins have been associated with FTD in previous CSF antibody-based proteomics (VEGFA [64]), or FTLD brain proteomics studies (ADAM23 [65–67] and WIF1 [68]). We also identified a subset of CSF proteins (30%) that were differentially regulated in both patients with FTD and AD compared to controls but with a different protein abundance between FTD and AD. Despite most of these being more prominently dysregulated in AD (e.g., SDC4, ITGB2, MIF, and sTREM1) [29, 69, 70], a small subset of proteins showed an opposite effect between these dementias (e.g., CHL1, GPC1, and CNTN5). Previous studies additionally detected decreased levels of CNTN5 and CHL1 in FTD and PSP using alternative platforms, which further supports the validity of our findings [71–73]. In addition, we detected a subset of proteins (20%) likely reflecting an overall neurodegenerative process, as they were similarly dysregulated in AD and FTD compared with controls. For instance, CHIT1 and MMP10 protein levels were increased in FTD and AD, as reported previously [29, 64, 74–79]. This study identifies numerous CSF proteins dysregulated in FTD. However, due to the inclusion of an AD group, we uncovered that half (43 out of 92) of the proteins are specifically associated with FTD, highlighting the importance of comparing to both controls and similar neurological diseases in biomarker studies. Among the proteins specifically associated with FTD, the ones showing the strongest effects were: WIF1, MMP7, APP, NPDC1, and IL1RL2. WIF1 is an inhibitory protein involved in the Wnt signaling cascade, a dysregulation of this pathway has been implicated in tau phosphorylation and other neuronal processes (e.g., neurogenesis, synaptic health and plasticity) [80, 81]. In addition, WIF1 was dysregulated in FTLD frontal cortex tissue [68], supporting its role within FTD pathophysiology, however, its specific function in relation to FTD remains to be elucidated. Enrichment analysis showed that proteins specifically dysregulated in FTD were enriched in biological pathways associated with axon development, regulation of synapse assembly, and cell-cell adhesion mediator activity. This is in line with previous unbiased brain and CSF proteomics studies, and multiplex CSF analysis showing a dysregulation of similar pathways in FTLD [31, 65, 66, 82, 83].

Considering that biomarkers to discriminate the FTLD-subtypes are currently lacking. We additionally investigated the CSF proteome in the main FTLD pathological subtypes. Despite many CSF proteins that were dysregulated in FTLD-Tau (60 proteins) or FTLD-TDP (120 proteins) compared to controls, overlapping analysis across all comparisons revealed three proteins related to FTLD-Tau (VSIG4, Siglec9, and CD84), two proteins related to FTLD-TDP (GRN and C1QTNF1), and one protein uniquely changed between the neuropathological subtypes (COCH). Given the established association between *GRN* mutations and TDP pathology, the decrease of GRN (progranulin) protein levels observed in FTLD-TDP aligns with previous GRN reductions in CSF and plasma of FTD-*GRN* mutation carriers [84, 85]. It should be noted that the FTLD-TDP group included a low number of *GRN* mutation carriers (11%). Considering that we observed decreased GRN levels in the total FTLD-TDP group, these results highlight that lysosomal dysfunction is an important feature in TDP pathology, which is additionally supported by post-mortem analysis [86, 87]. Furthermore, we also identified novel markers such as VSIG4. This protein was also found to be elevated in *MAPT* mutation carriers compared to non-carriers in a recent MS-proteomics study [88]. As a phagocytic receptor, it plays a key role in inflammatory responses and can mediate activation of the NLRP3 inflammasome [58]. Since the NLRP3 inflammasome plays a role within tau pathophysiology, this protein seems promising to reflect ongoing activation of the NLRP3 inflammasome in FTD patients [89]. To the best of our knowledge, many of the subtype-specific proteins identified in this study are novel and have not yet been linked to the main FTD pathologies or genetic subtypes in previous proteomics studies [30, 31, 65, 66]. Thus, these proteins require further investigation in CSF and brain tissue, to examine their relevance to ongoing tau or TDP43 pathophysiologies.

Next, we identified a classification signature of 10 CSF proteins to discriminate the two main FTLD subtypes (AUC: 0.8), with higher performance than the pTau/tTau ratio (AUC: 0.68). However, the moderate performance together with the large confidence intervals suggests that the model is rather unstable and may vary depending on the samples included. This variation could be explained by the inclusion of different FTD biochemical profiles, which likely differ between sporadic and familial cases with the same proteopathy, or even between genetic backgrounds of the same pathological subtypes (e.g., *GRN* and *C9orf72*) [14, 71, 88, 90]. Despite the unprecedented number of FTLD samples and proteins analyzed in this study, we identified relatively few robust markers associated with specific pathological subtypes. This limited discriminative power underscores both the biological complexity of FTLD and the inherent difficulty of distinguishing subtypes in vivo, a challenge that remains a critical hurdle for the field. While previous studies have reported promising results in differentiating broader diagnostic categories such as AD and FTD, the finer stratification of the FTLD subtypes requires deeper molecular insights. Notably, we observed a number of proteins with nominally significant differences across the FTLD subtypes, suggesting that larger, more powered studies may reveal additional subtype-specific biomarkers. These findings also reflect the heterogeneity within each FTLD subtype, emphasizing the importance of analyzing well-characterized cohorts, ideally with pathological and/or genetic confirmed individuals [14].

To translate the CSF proteome findings into practical biomarker tools for routine diagnostics or clinical trials, we applied classification analyses and identified two panels of 14 and 13 CSF proteins that can discriminate FTD from controls and AD dementia with high accuracies (AUCs of 0.96 and 0.91, respectively). Thereafter, custom multiplex assays were developed and validated in several independent cohorts. The fold changes for each protein correlated well between discovery and validation cohorts (*Rho’s* between 0.45 and 0.93). The high discriminative values for FTD were confirmed in three cohorts for both panels (AUCs > 0.877), supporting the robustness of our findings. In addition, these panels show added value by identifying FTD mutation carriers approaching symptom onset, and could thus be relevant to detect early pathological changes. Noteworthy, our novel FTD panels have similar discriminative performance compared to NfL. However, while NfL reflects neuroaxonal damage, our CSF panels offer a more comprehensive depiction of the underlying biological processes in FTD. The relevance of these FTD panels is additionally supported by the association of some markers (e.g., MMP10, PLTP, PRDX1, and NPDC1) with clinical parameters such as cognitive functioning or disease severity. For example, we detected positive correlations of APEX, PRDX1, and CHIT1 with FTLD-CDR scores. These proteins are related to oxidative stress and reactive microglial activity, processes that underlie FTD pathogenesis, and may therefore be relevant for disease staging [91, 92]. In addition, in agreement with previous studies, we observed that increased MMP10 levels were associated with worse cognitive performance [29, 74]. This could potentially be explained by the expression of MMP10 by microglial cells in the brain, as elevated MMP10 levels contribute to ongoing inflammatory responses leading to axonal damage, potentially leading to functional deficits such as cognitive impairment [93]. Overall, these associations provide further support for the potential value of these panels in clinical settings and trial contexts.

This study is not without limitations. The targeted proteomic approach employed in this study misses relevant proteins that might have been measured using unbiased MS methods (e.g., GFAP and neuropentraxins). However, our study still covered > 600 proteins covering a wide range of biological mechanisms and the workflow employed allowed us to swiftly translate our proteomic discovery findings into custom immunoassays for subsequent validation. Noteworthy, potential FTD misdiagnosis may influence biomarker results. However, diagnoses were made in specialized memory clinics and 47% of FTD individuals were either pathologically or genetically confirmed, and results were further validated in multiple independent cohorts. Furthermore, we observed that a subset of confirmed FTD patients in the discovery cohort exhibited a positive AD biomarker profile indicative of AD-co-pathology. Exclusion of these cases minimally affected the differential protein expression analyses, but increased the discriminative potential in the validation cohorts, which can probably be attributed to the association of several proteins in the panel with AD pathology (e.g., ABL1, THOP1, SMOC2, and ITGB2). Nevertheless, the potential influence of AD co-pathology on the FTD proteome should be explored in future studies involving larger sample sizes. Noteworthy, validation in the FTD genetic cohort demonstrated a high discriminative performance of our CSF biomarker panels in both presymptomatic and symptomatic FTD carriers compared to non-carriers. Although the smaller sample size in this cohort, relative to our other cohorts, may impact the reliability of the analysis, the strong performance of the panel remains promising for detecting FTD across different disease stages. However, these biomarker panels are not yet ready for clinical implementation. Additional validation in independent FTD cohorts is required to confirm their robustness and generalizability. Furthermore, it would be valuable to assess the performance of these novel panels in other disorders that share clinical and/or neuropathological features with FTD, such as primary psychiatric disorders or amyotrophic lateral sclerosis.

## Conclusions

This study identified CSF proteome changes specifically associated to FTD. We have translated these findings into CSF biomarker panels that can accurately discriminate FTD from controls and AD in multiple cohorts, and in the presymptomatic stage of the disease. Furthermore, we detect protein changes specifically associated to FTLD-Tau or FTD-TDP, although larger and more homogeneous cohorts will have more power to discriminate between these main pathological subtypes. The panels developed within this study could prove valuable for diagnosis or to monitor treatment effects in clinical trials. The antibody-based technology employed in this study allowed us to efficiently translate our discovery findings into custom multiplex panels for further clinical validation showing reproducible findings. This workflow applied here could also be helpful for the development of fluid biomarkers in other human matrices (e.g., blood) and other biomedical fields beyond neurodegenerative dementias.

## Supplementary Information

Below is the link to the electronic supplementary material.

Supplementary Material 1

Supplementary Material 2

Supplementary Material 3

Supplementary Material 4

Supplementary Material 5

Supplementary Material 6

Supplementary Material 7

## Data Availability

The source data generated in this study are available within this study (Supplementary Table 2, Supplementary Table 3, Supplementary Table 4, Supplementary Table 5). All models were built using publicly available packages and functions in R.

## Abbreviations

AD: Alzheimer’s dementia
ADC: Amsterdam dementia cohort
AUC: Area under the curve
CSF: Cerebrospinal fluid
CON: Cognitively normal individuals
CBS: Corticobasal syndrome
FDR: False discovery rate
FTD: Frontotemporal dementia
FTLD: Frontal temporal lobar degeneration
LOD: Lower limit of detection
MMSE: Mini–mental state examination
MS: Mass spectrometry
NPX: Normalized protein expression
PEA: Proximity extension assay
PPA: Primary progressive aphasia
PSP: Progressive supranuclear
QC: Quality controls
