# Supplementary material for: Large-scale CSF proteome profiling identifies biomarkers for accurate diagnosis of frontotemporal dementia
Source: Mol Neurodegener. 2025 Aug 27;20:93. doi: 10.1186/s13024-025-00882-5 (PMC12392567; doi:10.1186/s13024-025-00882-5)
Supplement: Supplementary file 7 — Supplementary Material 7 [file 13024_2025_882_MOESM7_ESM.docx]

**Additional file 1.**


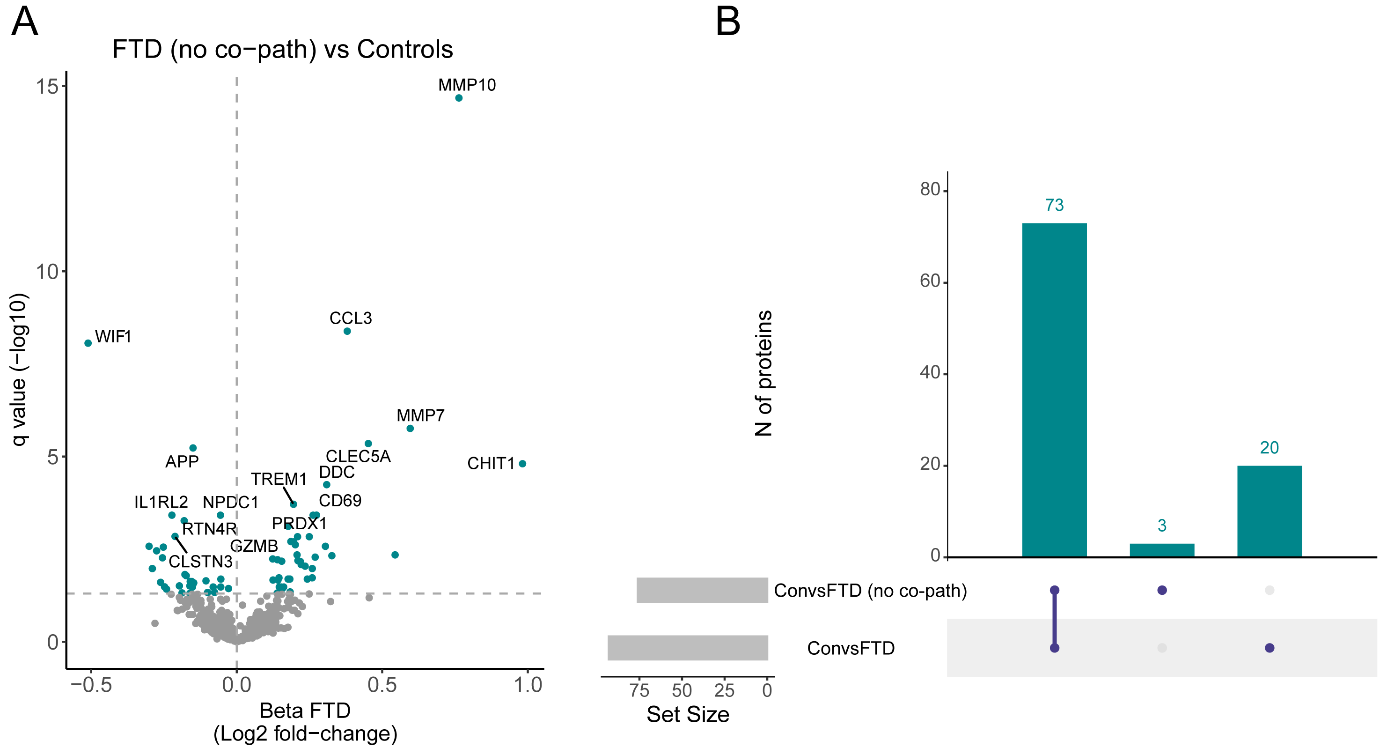


Figure 1. Differential abundance of CSF proteins in patients with FTD excluding AD co-pathology in the discovery cohort

Volcano plots show that 76 CSF proteins were differentially regulated between FTD patients with no AD co-pathology (n = 170) and controls (n = 196). Each dot represents a protein. The beta coefficients (log2 fold-change) are plotted versus q values (-log10-transformed). Proteins significantly dysregulated after adjusting for false discovery rate (FDR, q < 0.05) are depicted in blue. Horizontal dotted line indicate the significance threshold. UpSet plot shows proteins dysregulated between FTD patients with no AD co-pathology and controls and also dysregulated between FTD and controls.


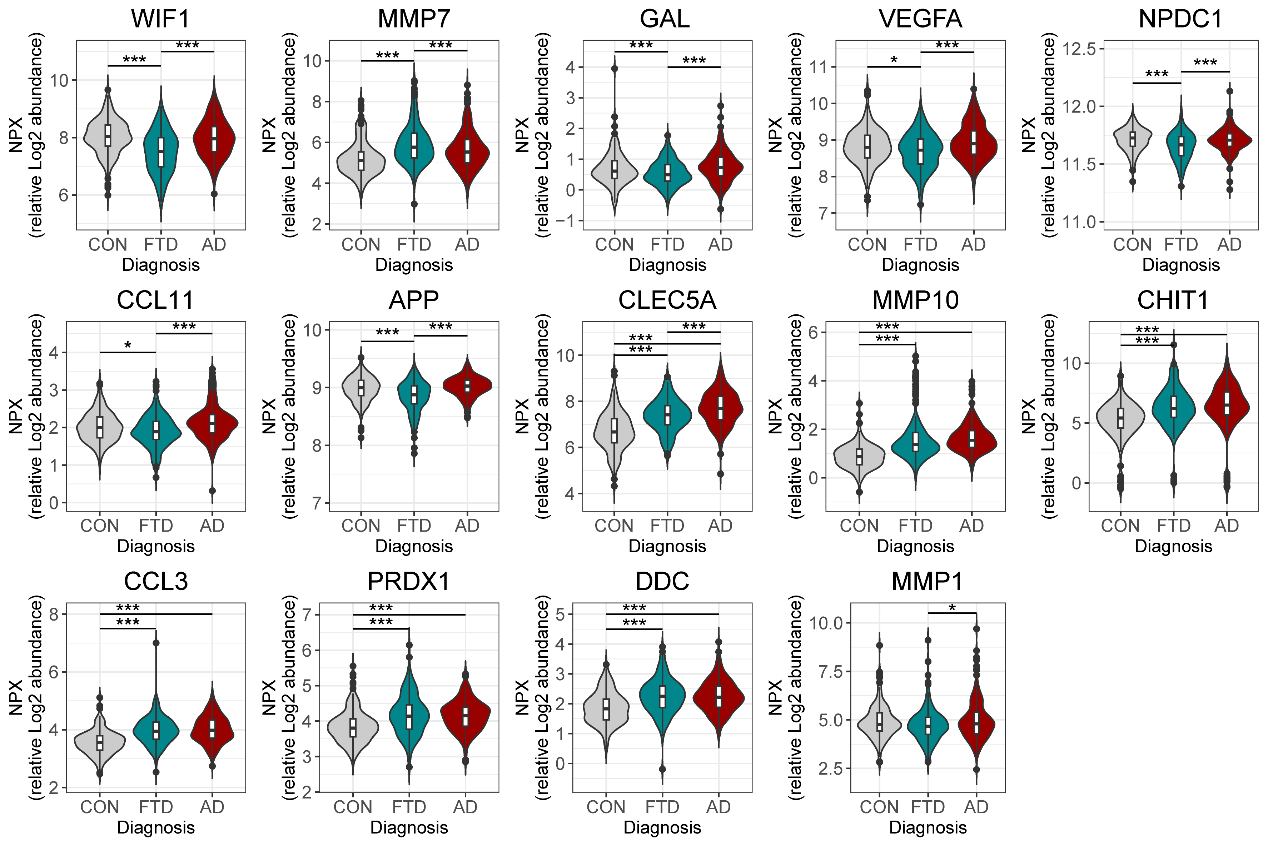


Supplementary Figure 2. Protein levels of FTD diagnostic biomarker panel

Violins represent the abundance (log2 NPX) of the CSF proteins that combined can accurately discriminate FTD from controls. Boxplot within the violin indicates the median and interquartile range of the protein abundance.*q < 0.05, **q < 0.01, ***q < 0.001. n.s: non-significant. Abbreviations: CON, cognitively unimpaired controls; FTD, frontotemporal dementia; AD, Alzheimer’s disease


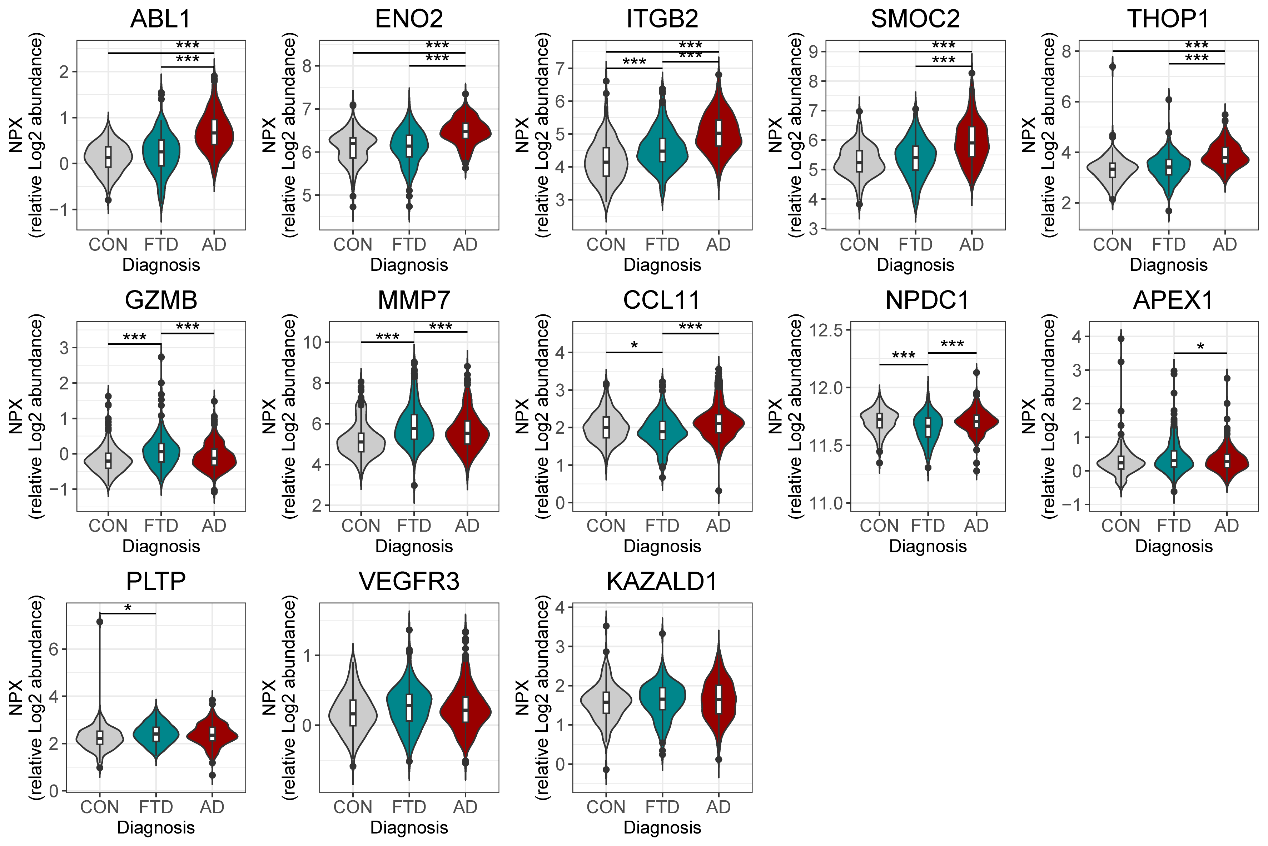


Supplementary Figure 3. Protein levels of FTD differential diagnostic biomarker panel

Violins represent the abundance (log2 NPX) of the CSF proteins that combined can accurately discriminate FTD from AD. Boxplot within the violin indicates the median and interquartile range of the protein abundance.*q < 0.05, **q < 0.01, ***q < 0.001. n.s: non-significant. Abbreviations: CON, cognitively unimpaired controls; FTD, frontotemporal dementia; AD, Alzheimer’s disease


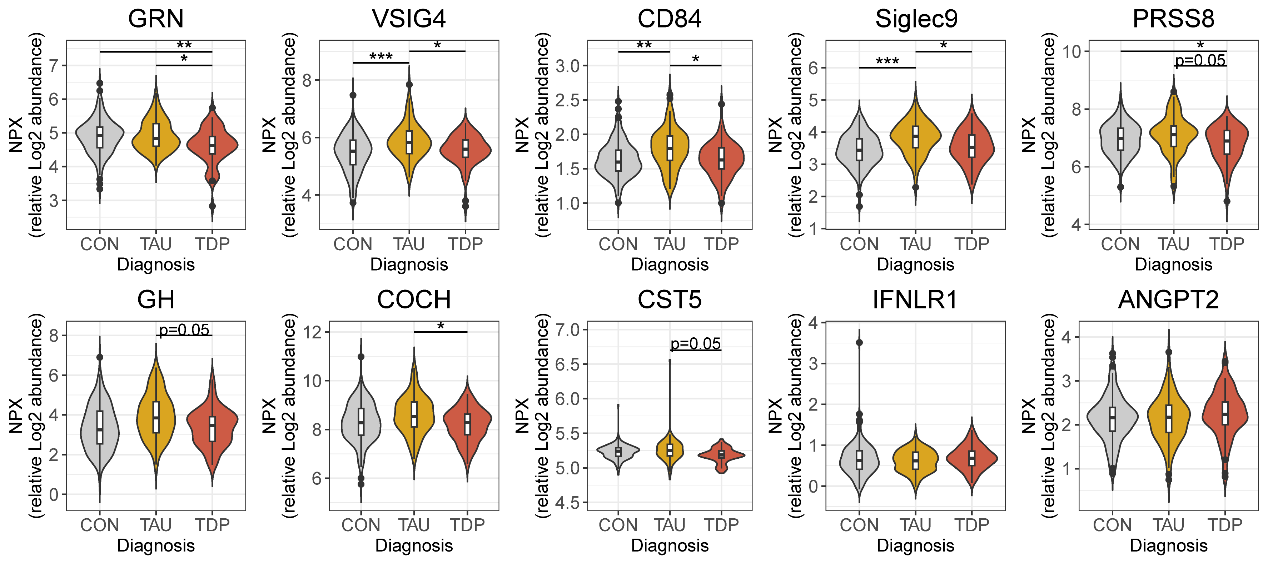


Supplementary Figure 4. Protein levels of FTLD subtype biomarker panel

Violins represent the abundance (log2 NPX) of the CSF proteins that combined can accurately discriminate FTLD-Tau from FTLD-TDP. Boxplot within the violin indicates the median and interquartile range of the protein abundance.*q < 0.05, **q < 0.01, ***q < 0.001. n.s: non-significant. Abbreviations: CON, cognitively unimpaired controls; TDP, Transactive response DNA binding protein of 43.


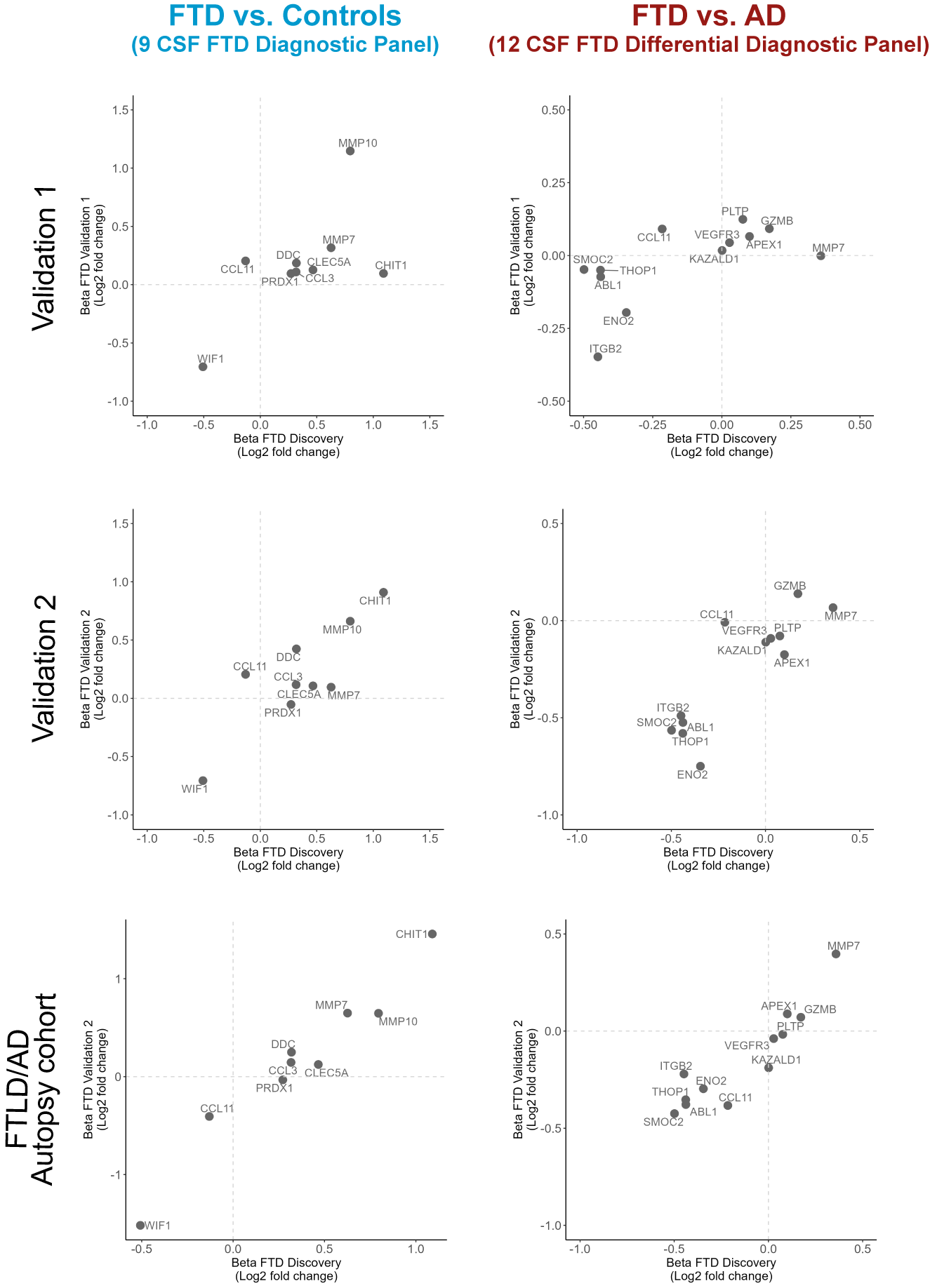


Supplementary Figure 5. Correlations between discovery and validation cohorts

Scatter plots depict the correlation between the beta-coefficients obtained in the discovery phase to those obtained with the custom assays in the clinical validation cohorts 1 and 2, and the FTLD/AD autopsy cohort.


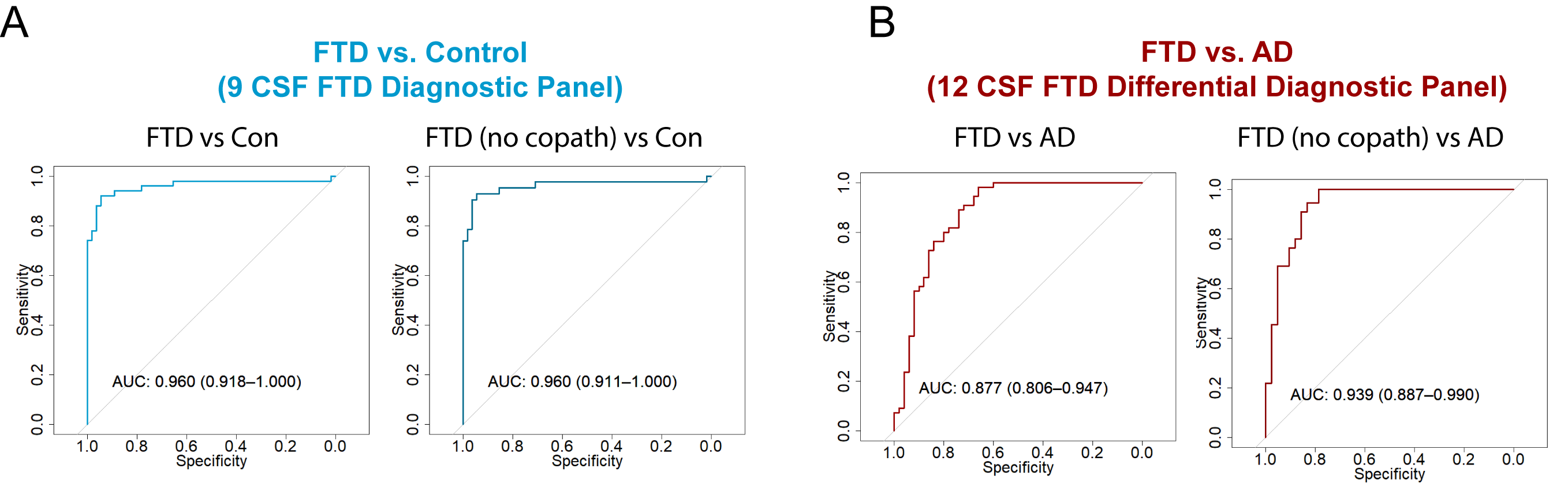


Supplementary Figure 6. Comparison of the performance of the CSF biomarker panels in FTD patients with and without AD co-pathology in validation cohort 1

Receiver operating characteristic (ROC) curves showing the performance of the CSF biomarker panel discriminating FTD from controls or AD using the custom assays in validation cohort 1. Inserts outline corresponding AUC and 95% CI.


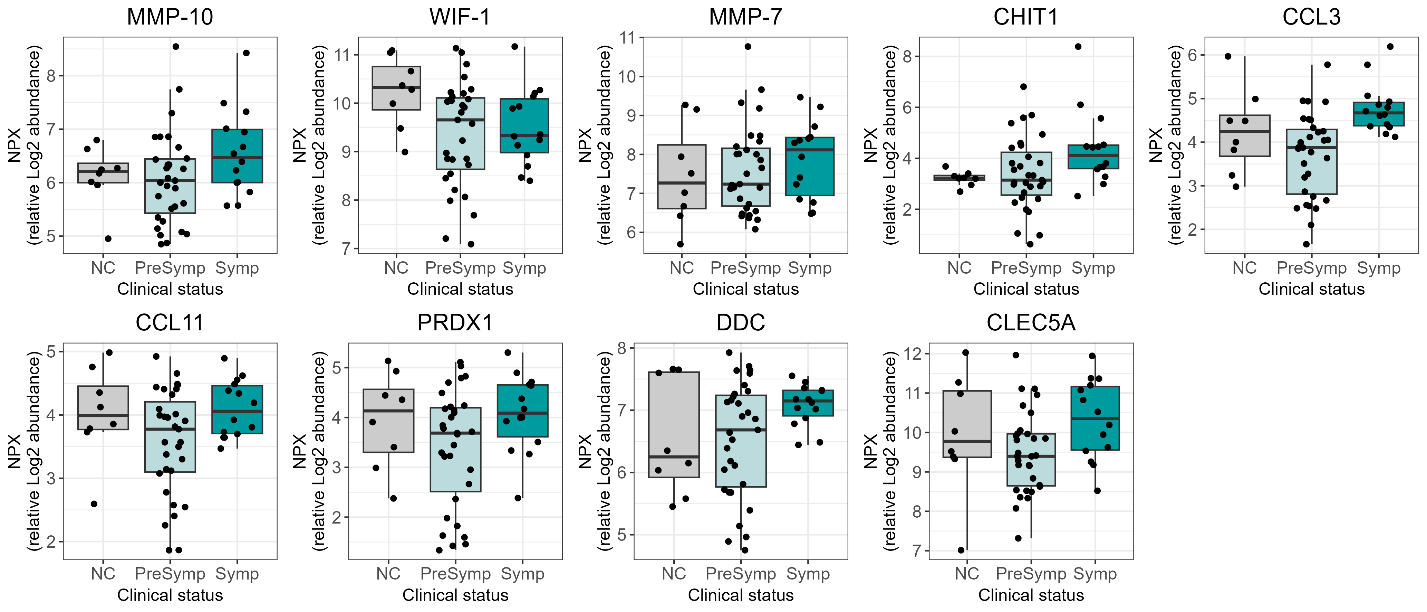


Supplementary Figure 7. Protein levels of the FTD diagnostic biomarker panel measured by the custom panel in the genetic FTD cohort

Boxplots represent the abundance (log2 NPX) of the CSF proteins that combined can accurately discriminate FTD from controls. Boxplot indicate the median and interquartile range of the protein abundance.*q < 0.05, **q < 0.01, ***q < 0.001. n.s: non-significant. Abbreviations: NC, non-carriers; PreSymp, Presymptomatic; Symp, Symptomatic FTD carriers.


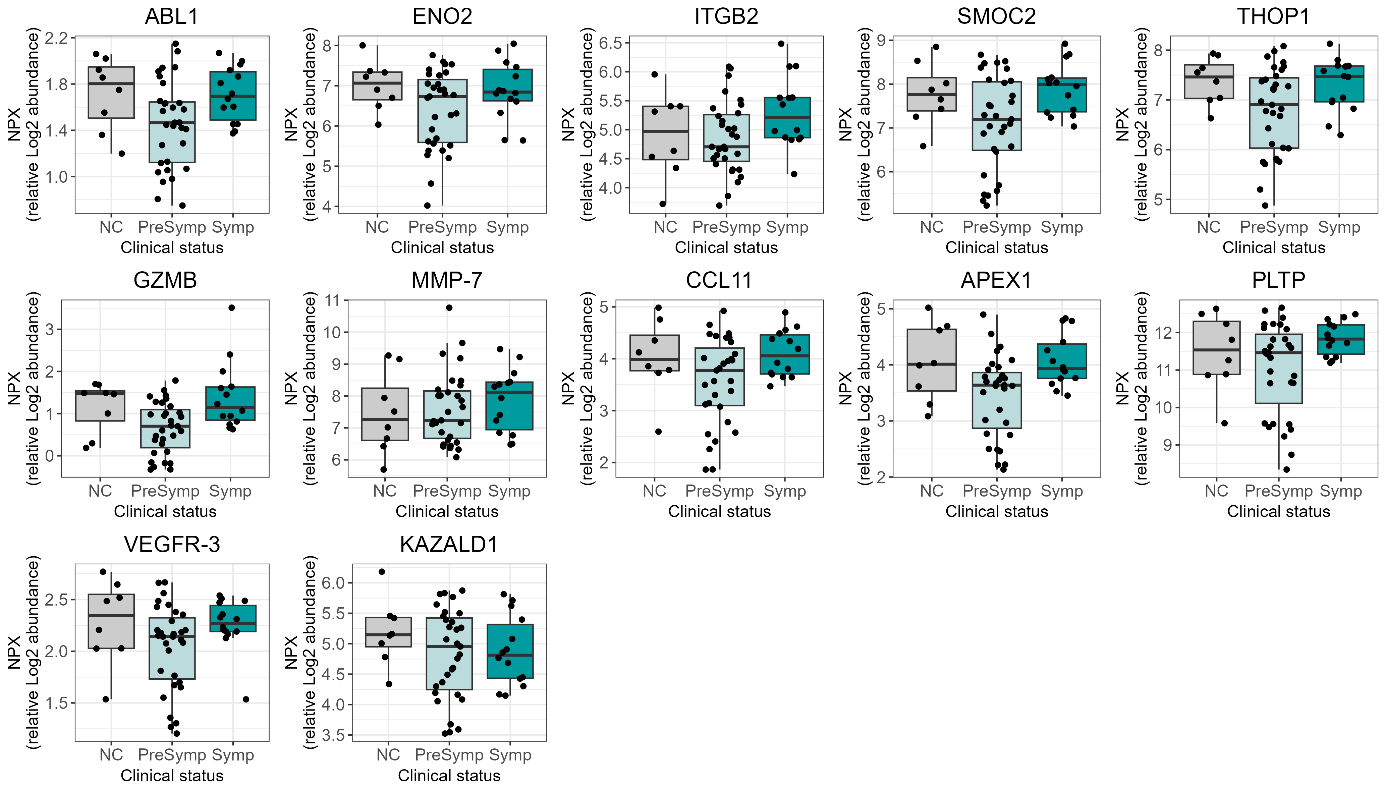


Supplementary Figure 8. Protein levels of the FTD differential diagnostic biomarker panel measured by the custom panel in the genetic FTD cohort

Boxplots represent the abundance (log2 NPX) of the CSF proteins that combined can accurately discriminate FTD from controls. Boxplot indicate the median and interquartile range of the protein abundance.*q < 0.05, **q < 0.01, ***q < 0.001. n.s: non-significant. Abbreviations: NC, non-carriers; PreSymp, Presymptomatic; Symp, Symptomatic FTD carriers.
